# Recent advances on halohydrin dehalogenases—from enzyme identification to novel biocatalytic applications

**DOI:** 10.1007/s00253-016-7750-y

**Published:** 2016-08-08

**Authors:** Anett Schallmey, Marcus Schallmey

**Affiliations:** Technische Universität Braunschweig, Institut für Biochemie, Biotechnologie und Bioinformatik, Spielmannstr. 7, 38106 Braunschweig, Germany

**Keywords:** Halohydrin dehalogenase, Dehalogenation, Epoxide ring opening, Protein engineering, Biocatalysis

## Abstract

Halohydrin dehalogenases are industrially relevant enzymes that catalyze the reversible dehalogenation of vicinal haloalcohols with formation of the corresponding epoxides. In the reverse reaction, also other negatively charged nucleophiles such as azide, cyanide, or nitrite are accepted besides halides to open the epoxide ring. Thus, novel C-N, C-C, or C-O bonds can be formed by halohydrin dehalogenases, which makes them attractive biocatalysts for the production of various β-substituted alcohols. Despite the fact that only five individual halohydrin dehalogenase enzyme sequences have been known until recently enabling their heterologous production, a large number of different biocatalytic applications have been reported using these enzymes. The recent characterization of specific sequence motifs has facilitated the identification of novel halohydrin dehalogenase sequences available in public databases and has largely increased the number of recombinantly available enzymes. These will help to extend the biocatalytic repertoire of this enzyme family and to foster novel biotechnological applications and developments in the future. This review gives a general overview on the halohydrin dehalogenase enzyme family and their biochemical properties and further focuses on recent developments in halohydrin dehalogenase biocatalysis and protein engineering.

**Electronic supplementary material:**

The online version of this article (doi:10.1007/s00253-016-7750-y) contains supplementary material, which is available to authorized users.

## Introduction

**Fig. 1:**
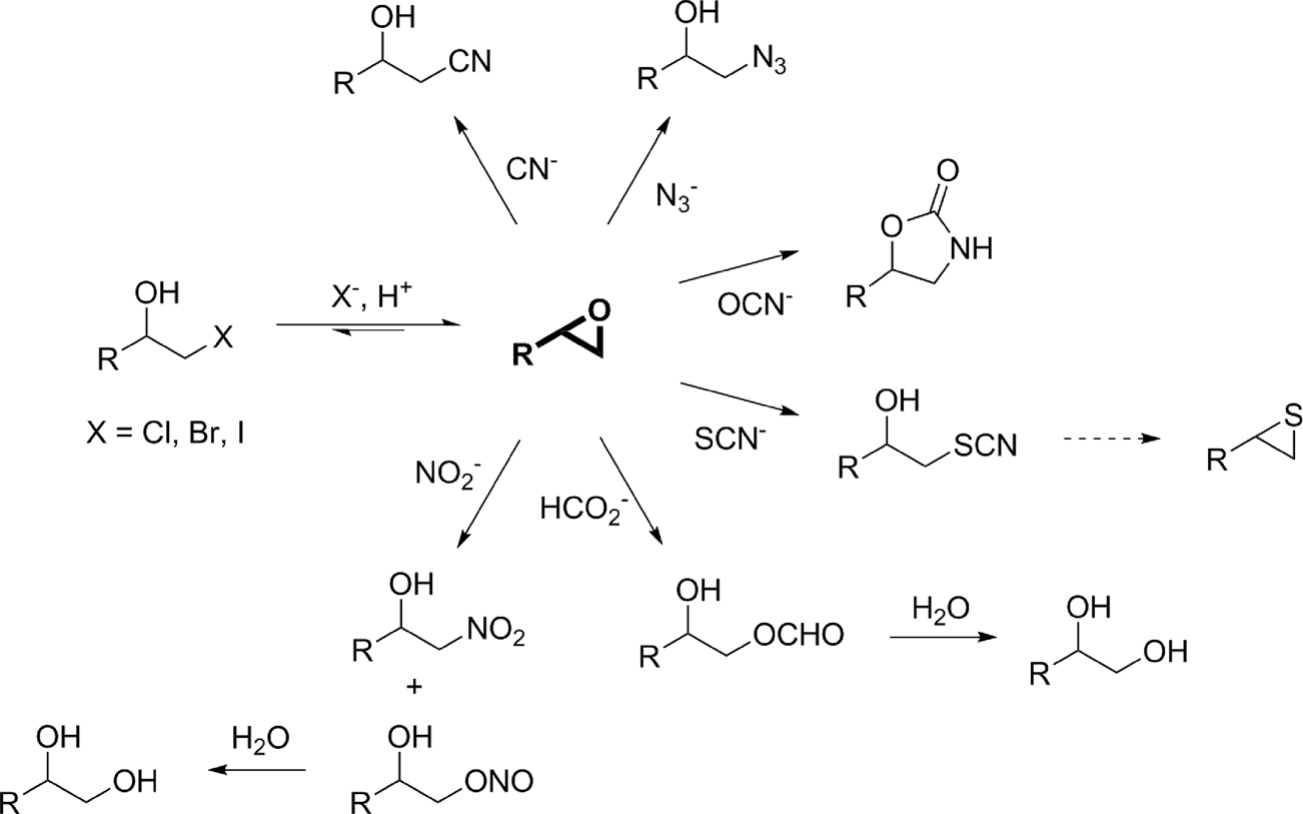
Catalytic scope of halohydrin dehalogenases in dehalogenation and epoxide ring opening

In 1968, Castro and Bartnicki reported for the first time an enzyme from *Flavobacterium* sp. involved in the degradation of 2,3-dibromo-1-propanol by removal of the bromo-substituent in 2-position and concomitant formation of the corresponding epoxide epibromohydrin (Castro and Bartnicki 1968). The enzyme was then termed halohydrin epoxidase and is member of a diverse group of dehalogenases (Janssen 2007). Since then, a number of enzymes able to dehalogenate various vicinal halohydrins have been isolated from different bacterial species and have been characterized for their biochemical and biocatalytic properties. These enzymes, also called halohydrin dehalogenases (HHDHs), haloalcohol dehalogenases, or halohydrin hydrogen-halide-lyases, belong to the enzyme class of lyases (EC 4.5.1.-) and catalyze the reversible dehalogenation of halohydrins with epoxide formation through intramolecular nucleophilic displacement of the halogen substituent by the neighboring hydroxyl group (Fig. 1). In addition, the enzymes can also utilize other negatively charged nucleophiles such as cyanide, azide, or nitrite in an irreversible epoxide ring opening reaction, resulting in the formation of novel C-C, C-N, or C-O bonds (Fig. 1; Hasnaoui-Dijoux et al. 2008). This reaction and nucleophile promiscuity of HHDHs, enabling the biocatalytic formation of various epoxides and β-substituted alcohols, makes them attractive candidates for practical application in biocatalysis.

Despite the rather large number of studies dealing with the isolation of microbial strains that exhibit HHDH activity and purification of the respective enzymes, only very few of the corresponding HHDH-encoding genes have been cloned. For that reason, until recently, only five different HHDH sequences have been published and assigned to one of the three different phylogenetic subtypes A, B, and C: *hheA* from *Corynebacterium* sp. strain N-1074 (Yu et al. 1994), *hheA2* from *Arthrobacter* sp. strain AD2 (van Hylckama Vlieg et al. 2001), *hheB* from *Corynebacterium* sp. strain N-1074 (Yu et al. 1994), *hheB2* from *Mycobacterium* sp. strain GP1 (van Hylckama Vlieg et al. 2001), and two identical *hheC* sequences from *Agrobacterium radiobacter* AD1 (van Hylckama Vlieg et al. 2001) and *Rhizobium* sp. strain NHG3 (Higgins et al. 2005). Of these, HheC from *A. radiobacter* AD1 has been studied the most as this enzyme seems to be an exception due to its remarkable (enantio)selectivity (Koopmeiners et al. 2016).

Naturally, these and other dehalogenases are reported to be involved in the mineralization of halogenated compounds (Janssen et al. 2005). Interestingly, however, only two of the HHDH genes known today are part of an operon together with an epoxide hydrolase required for further metabolization of the epoxide formed during HHDH catalysis (Schallmey et al. 2014). Hence, it cannot be excluded that halohydrin dehalogenases might exhibit also other physiological functions.

## Structural and mechanistic aspects

**Fig. 2:**
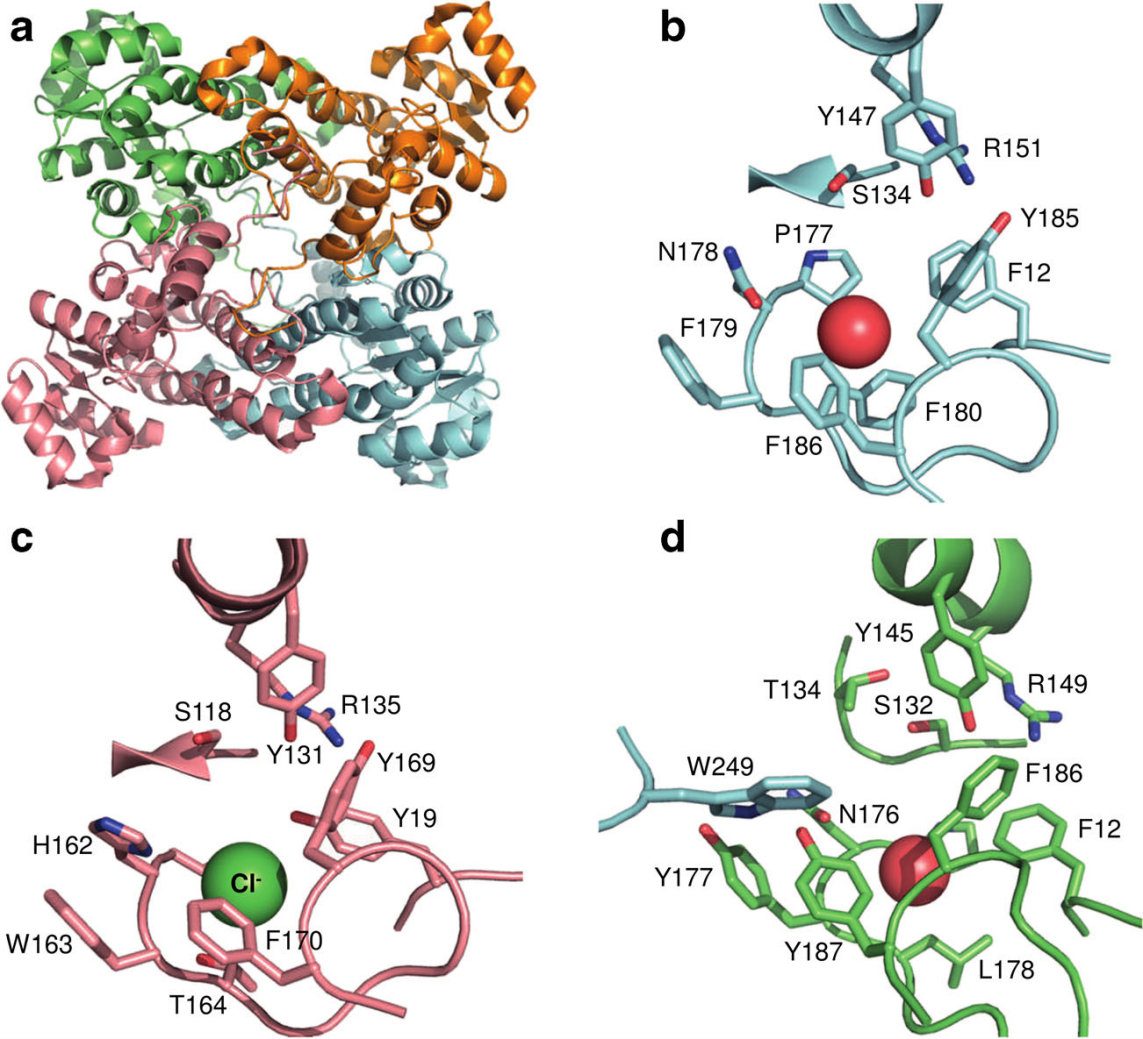
Structural aspects of halohydrin dehalogenases, **a** overall structure and tetrameric assembly of HheC as well as active-site structure of **b** HheA2 (PDB 1ZMO), **c** HheB (PDB 4ZD6), and **d** HheC (PDB 1ZMT). Water molecules in the nucleophile binding sites of HheA2 and HheC are shown as *red sphere*; the chloride ion in the active site of HheB is given as *green sphere*

**Fig. 3:**
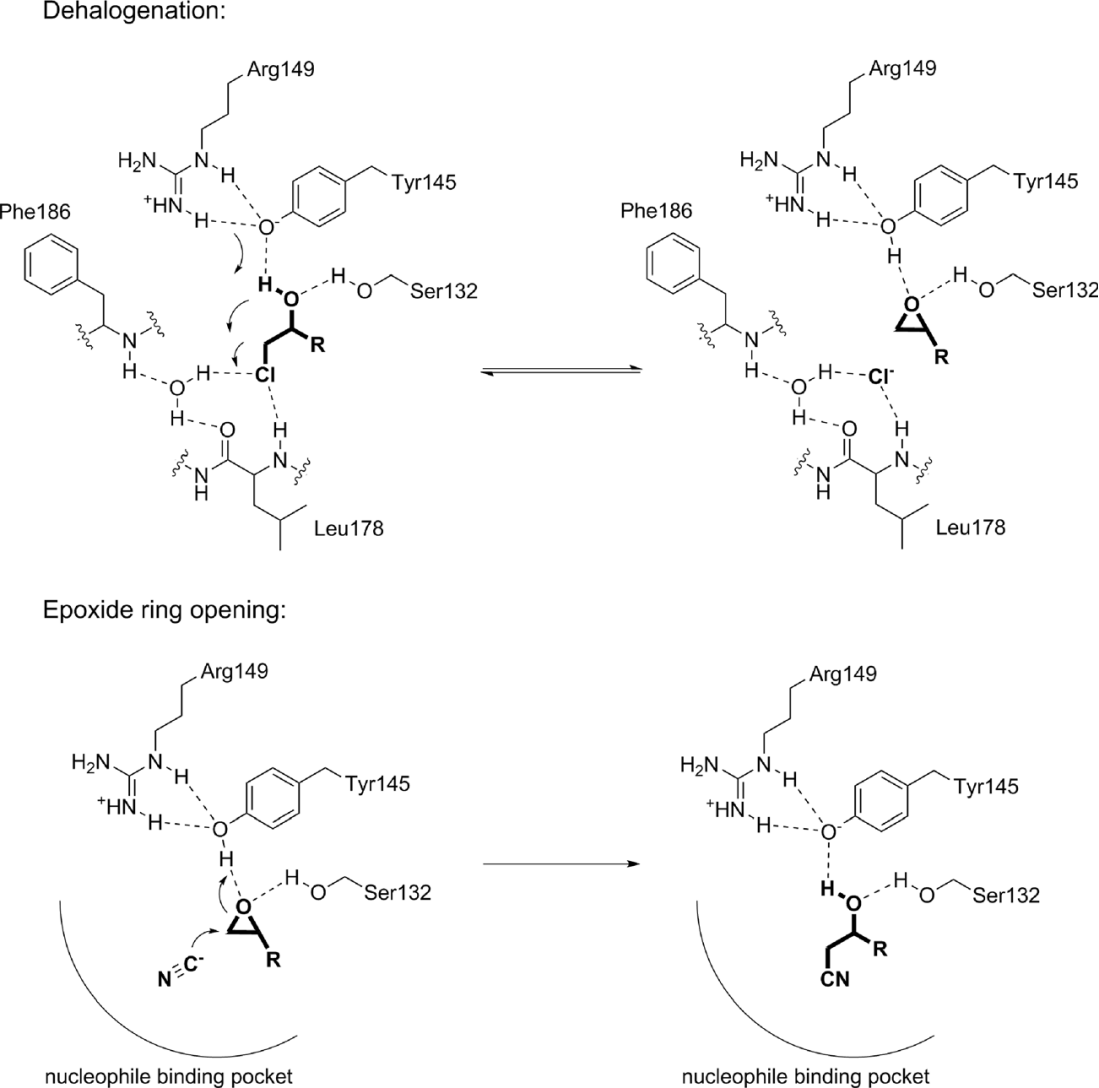
Catalytic mechanism of halohydrin dehalogenases in dehalogenation and epoxide ring opening; residue numbering according to HheC

Phylogenetically, HHDHs belong to the superfamily of short-chain dehydrogenases/reductases (SDRs), with which they share several structural and mechanistic features (van Hylckama Vlieg et al. 2001; Kavanagh et al. 2008). HHDHs are homotetramers, formed by a pair of dimers, and possess a catalytic triad made of Ser-Tyr-Arg similar to the Ser-Tyr-Lys catalytic triad of many SDR enzymes. Instead of the nicotinamide cofactor binding site found in SDR enzymes, a spacious nucleophile binding pocket is present at a corresponding position in HHDHs. To date, the crystal structures of HheA (Watanabe et al. 2015), HheA2 (de Jong et al. 2006), HheB (Watanabe et al. 2015), and HheC (de Jong et al. 2003) have been solved and reveal an overall highly similar structural fold. The monomers exhibit the typical Rossmann fold, also found for other SDR enzymes, consisting of a six- or seven-stranded parallel β-sheet that is surrounded by seven or eight α-helices (Fig. 2a). Each monomer’s active site is buried within the enzyme and is connected with the bulk solvent via a substrate entrance tunnel. This tunnel is differently shaped in the crystallized HHDHs and —at least for HheC— various studies demonstrate that residues of the entrance tunnel are involved in determining the enzymes’ activity and enantioselectivity (Schallmey et al. 2013; Guo et al. 2015; Wang et al. 2015b). The position of the catalytic residues Ser, Tyr, and Arg within the active sites is also highly conserved. During dehalogenation, the Ser residue positions the haloalcohol substrate within the active site by formation of a hydrogen bond with the substrate’s hydroxyl group, while the Tyr acts as a catalytic base abstracting a proton from that hydroxyl group (Fig. 3). The Arg residue does not interact with the substrate itself but lowers the pK_a_ of the tyrosine-OH via hydrogen bonding in order to activate it for proton abstraction (de Jong et al. 2003; Janssen et al. 2006; Schallmey et al. 2012). In the reverse reaction, i.e., epoxide ring opening, the Tyr donates a proton to the forming oxygen anion after nucleophilic attack of a halide (or another accepted nucleophile) on one of the carbons of the epoxide ring. The nucleophile binding pocket in HHDHs is formed by residues mapping to the glycine-rich T-G-X_3_-A/G-X-G motif of SDR enzymes as well as residues from other loops forming hydrogen bonds from backbone amides with the anionic nucleophile (de Jong et al. 2003). Additionally, a water molecule present in close proximity supports the hydrogen bond network around the nucleophile binding pocket.

Interestingly, only HheC possesses a C-terminal extension which protrudes into the active site of an opposing monomer within the tetrameric quaternary assembly. This C-terminal extension contains a Trp residue (Trp249; Fig. 2d), which significantly influences the stereoselectivity of HheC (Tang et al. 2003; de Jong et al. 2005).

## Enzyme family

For a long time, the previously cloned enzymes —HheA/A2, HheB/B2, or HheC— were the only representatives of the HHDH enzyme family. All these enzymes were obtained from bacterial strains enriched from environmental samples to degrade toxic compounds such as epihalohydrins or haloalcohols as sole carbon source (van den Wijngaard et al. 1989; Poelarends et al. 1999; Effendi et al. 2000) and sometimes yielded optically active C3 compounds (Nakamura et al. 1991). Although these individual bacterial strains were enriched from three different global sampling sites, the actual HHDH sequence identities were larger than 97 % for enzymes of the same HHDH subtype (Yu et al. 1994; van Hylckama Vlieg et al. 2001; Higgins et al. 2005). Further, using HHDH-specific oligonucleotides, only HHDHs with either 97 or 100 % enzyme sequence identity to HheA or HheC, respectively, could be obtained in recombinant form from related bacterial strains (Liu et al. 2014; Xue et al. 2014). Alternatively, public sequence databases are a common source for novel enzyme sequences, but direct identification of novel HHDHs via homology searches is hampered due to their actual high similarity to non-dehalogenating SDR enzymes. Due to sequence identities lower than 33 % between A-, B-, and C-type enzymes, conventional BLAST searches with any of the earlier described HHDHs as query commonly return better ranking SDR sequences before listing putative novel HHDH sequences. While it might be a viable option to recombinantly express and assay candidate sequences for true HHDH activity when only few other better ranking SDR hits exist in BLAST results (Wan et al. 2014), such experimental strategies are not practical for distantly related sequences with hundreds or thousands of possible candidate genes. To unambiguously identify true HHDHs from the large majority of SDR enzymes present in databases such as GenBank, HHDH-specific sequence motifs were recently derived from multiple sequence alignments of the known HHDHs to filter for candidate sequences with only target activity (Schallmey et al. 2014). Here, the combination of an N-terminal motif as part of the nucleophile binding pocket architecture (T-X_4_-F/Y-X-G) and a second motif resembling the HHDH catalytic triad (S-X_12_-Y-X_3_-R) was very effective to successfully identify 37 novel HHDH genes by PHI-BLAST. For a representative and diverse selection of 19 enzymes, true HHDH activity was confirmed after heterologous expression, illustrating the usefulness of this in silico identification strategy. Phylogenetic analysis revealed that several of these novel HHDHs could not be assigned to any of the previously classified subtypes A to C and thus required grouping within four additional subtypes D through G. Apparently, when characterizing 17 of these enzymes in more detail (Koopmeiners et al. 2016), this phylogenetic classification scheme seems to be largely in agreement with biocatalytic properties such as enantioselectivities. Although most of the selected 17 novel HHDHs exhibit only low sequence identities to previously known HHDHs, their biochemical and biocatalytic properties are still rather similar. Among these enzymes, only HheA3, HheA5, HheD, HheD3, and HheD5 stand out because they display higher thermostabilities and temperature optima in comparison to HheC. Enzymes HheA3 (Wan et al. 2014) and HheA5 (Xue et al. 2015a) from *Parvibaculum lavamentivorans* DS-1 and *Tistrella mobilis*, respectively, have been characterized by Zheng and coworkers as well. The authors reported a higher thermostability for HheA5 too as well as a high activity in the cascade-type conversion of ethyl (*S*)-4-chloro-3-hydroxybutyrate into ethyl (*R*)-4-cyano-3-hydroxybutyrate. Additionally, researchers around Zheng cloned and confirmed HHDH activity for an enzyme from *Agromyces mediolanus* (HheA12; Xue et al. 2014), an enzyme with 98 % sequence identity to previously known HheA4 (Schallmey et al. 2014).

**Fig. 4:**
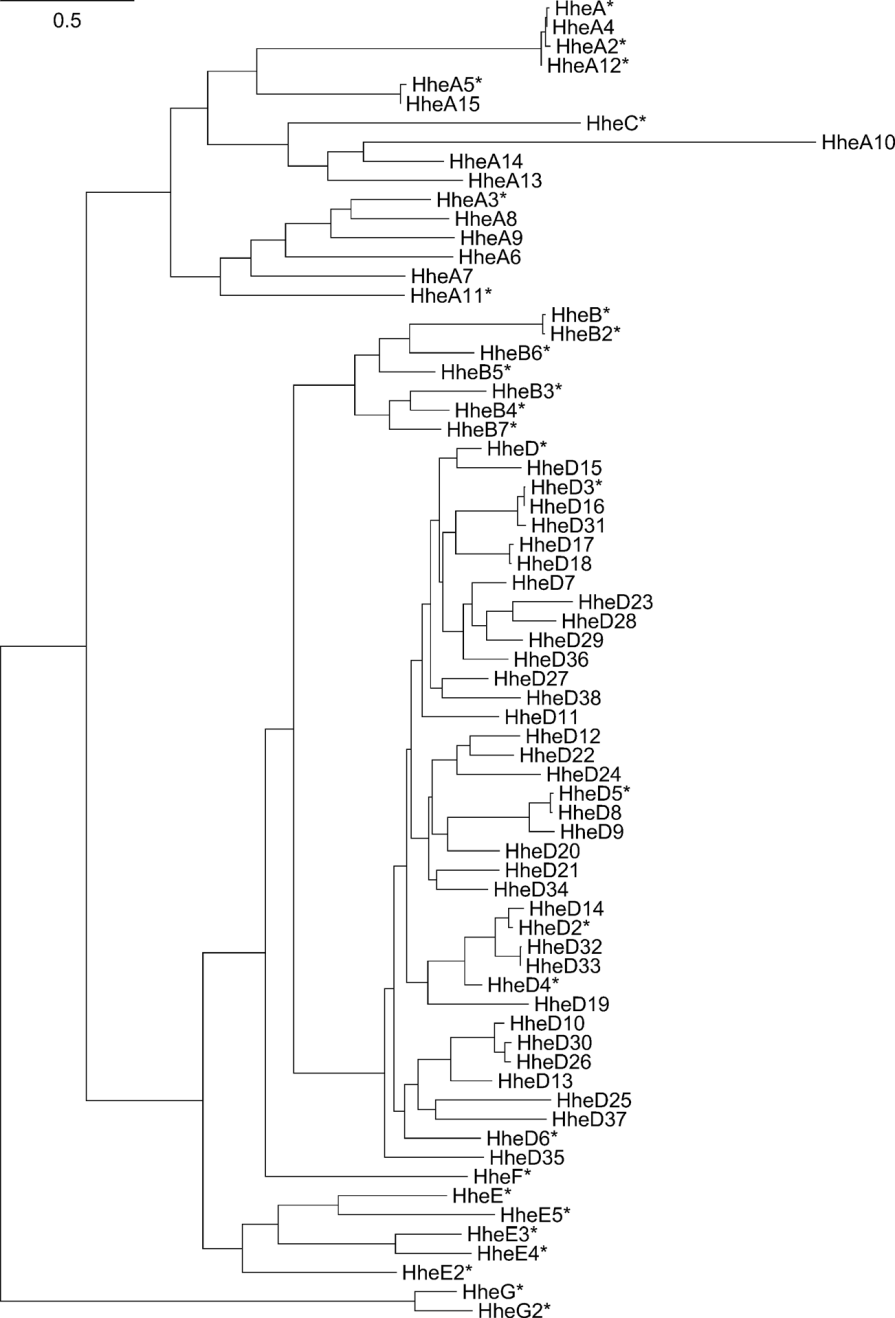
Phylogenetic representation of the extended HHDH enzyme family. The shown phylogram is constructed from a maximum likelihood built with W-IQ-TREE (Trifinopoulos et al. 2016) on the basis of a PRANK +F (Löytynoja and Goldman 2010) multiple sequence alignment of all HHDHs from Table S1. Experimentally verified HHDH sequences are marked by an *asterisk*

Applying both HHDH-specific sequence motifs in PHI-BLAST queries on the recent GenBank release (version 213), another 26 enzyme sequences can be identified belonging to one of the currently classified HHDH subfamilies and, thus, extend the growing list of HHDH enzymes (Table S1). Phylogenetically, most of these putative enzymes are either A- or D-subtype HHDHs (Fig. 4). Enzymes HheA10 and HheA13 through HheA15 are 27–42 % identical to HHDHs for which activity has been confirmed, including HheA11 (unpublished data) and HheA12 (Xue et al. 2014). In contrast to the more diverse branch of the A/C-type enzymes, HheD19 to HheD38 exhibit 57 to 95 % sequence identities to experimentally verified HHDHs HheD through HheD6.

## Biocatalytic applications

**Fig. 5:**
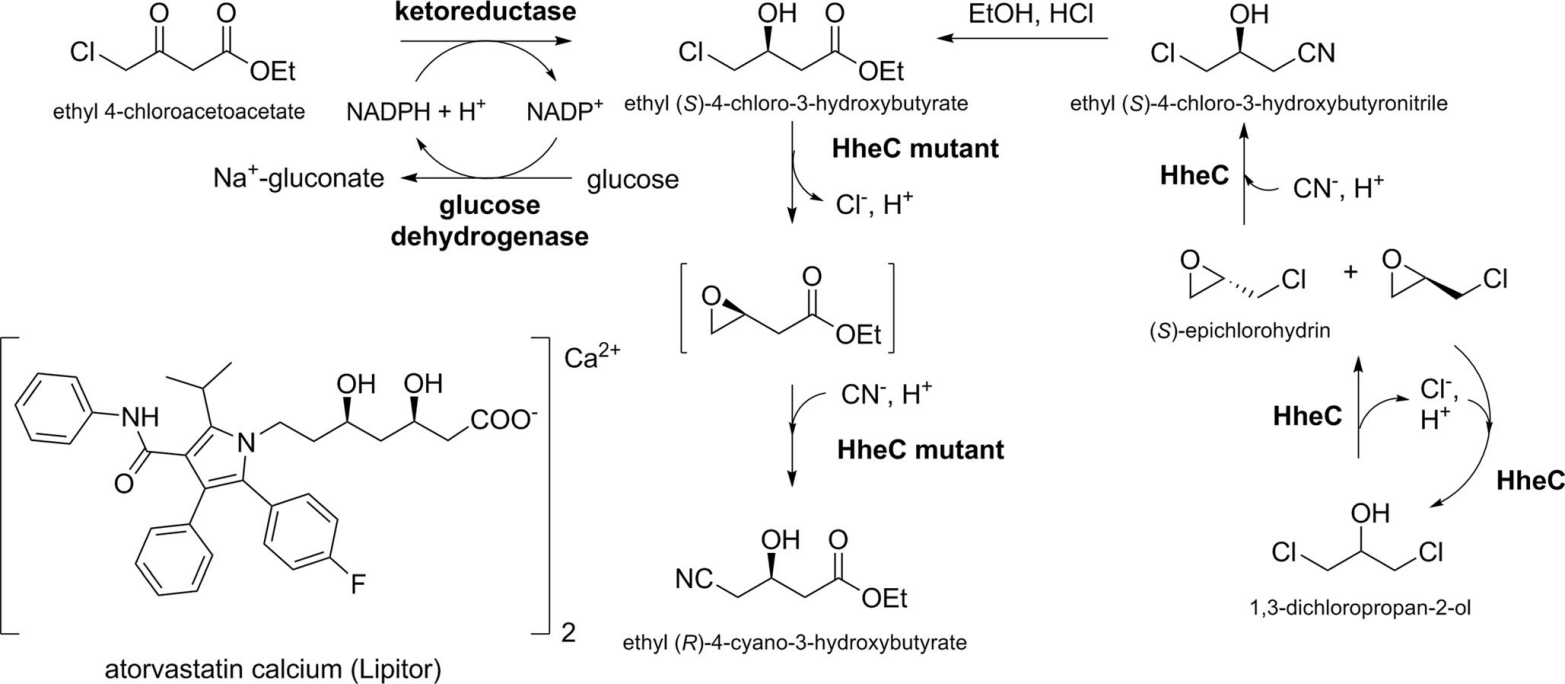
Biocatalytic synthesis of statin side-chain precursors

Various biotechnological applications, especially regarding stereoselective biotransformations, have been described using halohydrin dehalogenases. The most important one is certainly the production of the statin side-chain precursor ethyl (*R*)-4-cyano-3-hydroxybutyrate from ethyl (*S*)-4-chloro-3-hydroxybutyrate via the corresponding (*S*)-epoxide as intermediate (Fig. 5). This biocatalytic process starts from prochiral ethyl 4-chloroacetoacetate, which is selectively reduced by an (*S*)-selective ketoreductase affording ethyl (*S*)-4-chloro-3-hydroxybutyrate (Ma et al. 2010). The latter is further converted by a heavily engineered HheC mutant generated by ProSAR-based protein engineering to meet specific process requirements such as high cyanolysis activity or increased enzyme stability (Fox et al. 2007). Further biocatalytic applications of HHDHs include the synthesis of other enantiopure C3 or C4 units, such as epihalohydrins (Lutje Spelberg et al. 2004), or chiral tertiary alcohols (Majeric Elenkov et al. 2007; Fuchs et al. 2009). Besides that, several reports describe the use of HHDHs for the biodegradation of toxic xenobiotics such as 1,2,3-trichloropropanol present in contaminated soil and water (Dvorak et al. 2014) or the removal of 3-chloro-1,2-propanediol esters formed during food processing (Bornscheuer and Hesseler 2010). Very recently, also, the application of a halohydrin dehalogenase for the quantification of azide and cyanide in aqueous solution was reported (Wan et al. 2015c). The method is based on the HHDH-catalyzed reaction of azide or cyanide with 1,2-epoxybutane and detection of the resulting products 1-azidobutan-2-ol and 3-hydroxypentanenitrile, respectively, by gas chromatography.

Since surveying the HHDH literature in 2012, a number of papers on biocatalytic applications of HHDHs have been published. Only few of them, however, indeed present novel applications or synthetic routes toward new products. For a more complete picture, especially including earlier applications of HHDHs in biocatalysis and biotechnology in general, the reader is also referred to previous reviews (Janssen et al. 2006; Schallmey et al. 2012; You et al. 2012).

### Chiral C3/C4 units

Since the first synthesis of ethyl (*R*)-4-cyano-3-hydroxybutyrate, a precursor of atorvastatin, from ethyl-4-chloroacetoacetate by combination of an (*S*)-selective ketoreductase and an HheC mutant (Fig. 5; Fox et al. 2007), several recent biocatalytic studies on HHDHs have focused on the production of ethyl (*R*)-4-cyano-3-hydroxybutyrate. Wan et al. (2014) successfully applied HheA3 from *P. lavamentivorans* DS-1 in the preparative-scale synthesis of ethyl (*R*)-4-cyano-3-hydroxybutyrate starting from 200 g L^−1^ ethyl (*S*)-4-chloro-3-hydroxybutyrate using a whole-cell biocatalytic system. Within 14 h, 95 % conversion and 85 % yield could be reached, however, still employing a rather high catalyst loading (40-g L^−1^ lyophilized cells). An alternative route toward ethyl (*R*)-4-cyano-3-hydroxybutyrate proceeds via (*S*)-4-chloro-3-hydroxybutyronitrile (Fig. 5). Hence, production of (*S*)-4-chloro-3-hydroxybutyronitrile from cheap 1,3-dichloropropan-2-ol by HHDH catalysis was also investigated. This reaction proceeds via dehalogenation of the prochiral substrate and subsequent ring opening of the formed epoxide epichlorohydrin using cyanide as the nucleophile. Wan et al. (2015b) tested different HHDHs in this reaction and found that only HheC gave the desired (*S*)-4-chloro-3-hydroxybutyronitrile with sufficient enantiomeric excess. Here, stereoselectivity is introduced in the second reaction step as HheC preferentially converts (*S*)-epichlorohydrin to the corresponding butyronitrile. In the past, this high (*S*)-enantioselectivity of HheC has been already observed during azidolysis of epichlorohydrin (Lutje Spelberg et al. 2004). Now, by applying the more selective mutant HheC W249F, product ee could be further increased to >99 %. When scaling up the reaction to 200 mL, (*S*)-4-chloro-3-hydroxybutyronitrile was obtained in 86 % yield from 20 mM 1,3-dichloropropan-2-ol within 1 h, while the product ee decreased only slightly to 97.5 %.

Epichlorohydrin and other epihalohydrins are important intermediates in the preparation of polymers, adhesives, or pharmaceuticals and —especially for the latter— enantiomerically pure epihalohydrins are required (Kasai et al. 1998). Kinetic resolution of racemic epichlorohydrin by nitrite-mediated epoxide ring opening using HheC afforded (*R*)-epichlorohydrin with 99 % ee at 41 % conversion after only 18 min (Jin et al. 2012). Longer incubation times resulted in full conversion of the racemic epoxide as also the (*R*)-enantiomer is accepted by HheC, however, at a lower rate. Likewise, the authors investigated the one-pot, two-step synthesis of (*R*)-epichlorohydrin starting from prochiral 1,3-dichloropropan-2-ol. In this reaction, dehalogenation of 1,3-dichloropropan-2-ol and epoxide ring opening of the resulting epichlorohydrin were carried out in two sequential steps in order to adjust the reaction conditions to optimal pH and temperature in between. This was required to yield (*R*)-epichlorohydrin in high (99 %) enantiomeric excess, although yield (25 % starting from the chloroalcohol) was still low. In another approach, Jin et al. (2013) were able to synthesize (*R*)-epichlorohydrin with an ee of 99 % by combining HheC with an epoxide hydrolase in a two-phase reaction system.

Besides that, Zheng and coworkers published several papers dealing with the production of epichlorohydrin, either racemic or enantiomerically pure, as well as (*S*)-2,3-dichloro-1-propanol by HHDH catalysis, applying different process optimization strategies such as in situ product removal, catalyst immobilization, and/or organic-aqueous two-phase systems (Zou et al. 2013; Zou et al. 2014).

### Cascade reactions

**Fig. 6:**
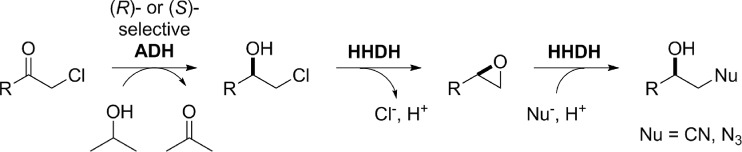
Biocatalytic synthesis of chiral β-substituted alcohols by combination of a stereoselective alcohol dehydrogenase (ADH) and a halohydrin dehalogenase (HHDH) (Schrittwieser et al. 2009)

In the last years, biocatalytic cascade reactions have emerged as powerful tool for the synthesis of various products. By combination of different enzymatic reaction steps in one pot and in analogy to catalysis performed in nature, intermediate product workup is omitted and reaction equilibria can be shifted leading to an improved process efficiency and reduced costs. In the field of HHDH catalysis, this cascade principle has already been applied very early by combining HheC with an epoxide hydrolase for the synthesis of optically active halohydrins, epoxides, and diols (Lutje Spelberg et al. 1999). Since then, further enzymatic cascade reactions employing HHDHs have been reported including the synthesis of enantiopure β-azidoalcohols and β-hydroxynitriles starting from prochiral α-chloroketones by combining an HHDH with (enantiocomplementary) alcohol dehydrogenases (ADHs; Fig. 6) (Schrittwieser et al. 2009; Szymanski et al. 2010). Recently, Chen et al. (2013) developed this concept further by coexpressing all necessary enzymes (ADH, HHDH, and cofactor-regenerating enzyme) in one bacterial host. By adjusting expression levels of the different enzymes and optimizing cofactor recycling, reaction times could be significantly reduced while still reaching full conversion of the tested chloroketones to the final products.

Zhu and coworkers reported a very efficient one-pot process for the production of ethyl (*R*)-3-hydroxyglutarate from ethyl (*S*)-4-chloro-3-hydroxybutyrate using recombinant *Escherichia coli* whole cells (co)expressing an HheC mutant and nitrilase AtNIT2 from *Arabidopsis thaliana* (Yao et al. 2015). This cascade involves three reaction steps starting with the dehalogenation of ethyl (*S*)-4-chloro-3-hydroxybutyrate and subsequent ring opening of the epoxide intermediate using cyanide as the nucleophile catalyzed by the HHDH, followed by hydrolysis of the introduced nitrile group catalyzed by nitrilase AtNIT2. Using *E. coli* whole cells coexpressing both genes, 600 mM ethyl (*S*)-4-chloro-3-hydroxybutyrate was completely converted into ethyl (*R*)-3-hydroxyglutarate within 6 h. As the nitrilase was inhibited by ethyl (*S*)-4-chloro-3-hydroxybutyrate concentrations above 300 mM and NaCN concentrations above 200 mM, batch feeding of the haloalcohol substrate as well as cyanide feeding to control the pH of the reaction around pH 8–9 was applied. Performing the HHDH and nitrilase reactions as one-pot, two-step process —i.e., addition of nitrilase-expressing cells after full conversion of ethyl (*S*)-4-chloro-3-hydroxybutyrate to ethyl (*R*)-4-cyano-3-hydroxybutyrate— even allowed to run the reaction at 1.2 M substrate concentration with complete conversion within 12 h.

### Spiroepoxides and oxazolidinones

**Fig. 7:**
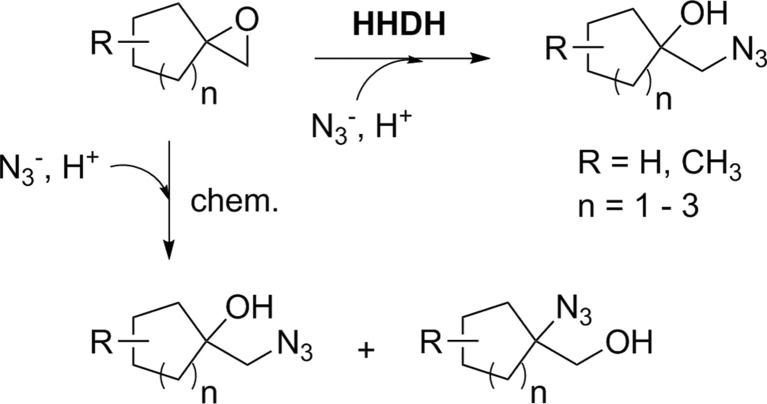
HHDH-catalyzed azidolysis of spiroepoxides (Majeric Elenkov et al. 2012)

Recently, HHDHs were shown to also convert spiroepoxides. Azidolysis of different chiral and achiral spiroepoxides containing five- to seven-membered cycloalkane rings afforded the corresponding β-azidoalcohols, carrying the OH group at the tertiary carbon atom, in a highly regioselective fashion when using HheA2 or HheC (Fig. 7; Majeric Elenkov et al. 2012). This is in contrast to chemical azidolysis of spiroepoxides which results in the formation of regioisomeric mixtures. Using HheC, reactions of chiral spiroepoxide substrates even proceeded with moderate to high enantioselectivity (*E* = 21–200), whereas enantioselectivity of HheA2 was only low (*E* ≤ 3). The obtained experimental results were further supported by quantum-chemical calculations and docking simulations explaining the regioselectivity and stereoselectivity of both enzymes.

**Fig. 8:**
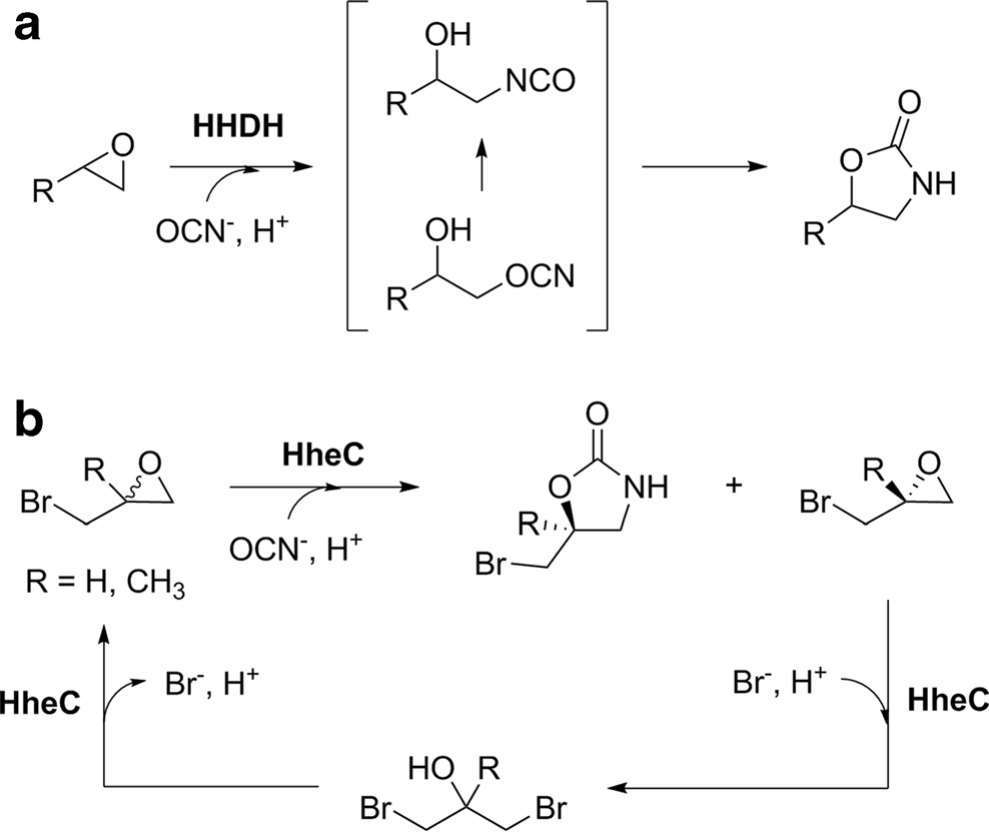
Synthesis of chiral oxazolidinones by HHDH-catalyzed epoxide ring opening with cyanate, **a** general reaction principle and **b** dynamic kinetic resolution of racemic epibromohydrins for the production of the corresponding (*S*)-oxazolidinones (Mikleusevic et al. 2015)

Previously, Majeric Elenkov et al. (2008) reported the synthesis of enantiopure five-substituted oxazolidinones by HheC-catalyzed kinetic resolution of different aliphatic epoxides. Epoxide ring opening with cyanate as nucleophile initially affords the corresponding isocyanate-cyanate species which undergo rapid cyclization resulting in the formation of 2-oxazolidinones as final products (Fig. 8). This concept was now extended to a dynamic kinetic resolution by in situ racemization of the slower reacting epoxide enantiomer through reversible epoxide ring opening with bromide, also catalyzed by HheC (Fig. 8; Mikleusevic et al. 2015). Thus, starting from epibromohydrin and 2-bromomethyl-2-methyl-oxirane, the corresponding (*S*)-configured oxazolidinones were obtained in high yield (97 and 87 %, respectively) and high optical purity (89 and >99 % ee, respectively) using only catalytic amounts of NaBr. Especially, (*S*)-oxazolidinones are important structural components of pharmaceuticals such as oxazolidinone antibiotics (Barbachyn and Ford 2003) and are important chiral auxiliaries and ligands in chemical synthesis (Ager et al. 1996).

## Assay developments

Over the years, different, usually spectrophotometric assays to quantify HHDH activity have been reported in literature. Initially, these assays have been developed to measure and compare enzyme activity and kinetics of purified HHDHs in a simple and fast way. Over the last decade, however, such assays have become more and more important for HHDH engineering to screen corresponding mutant libraries for new variants with desired characteristics.

**Fig. 9:**
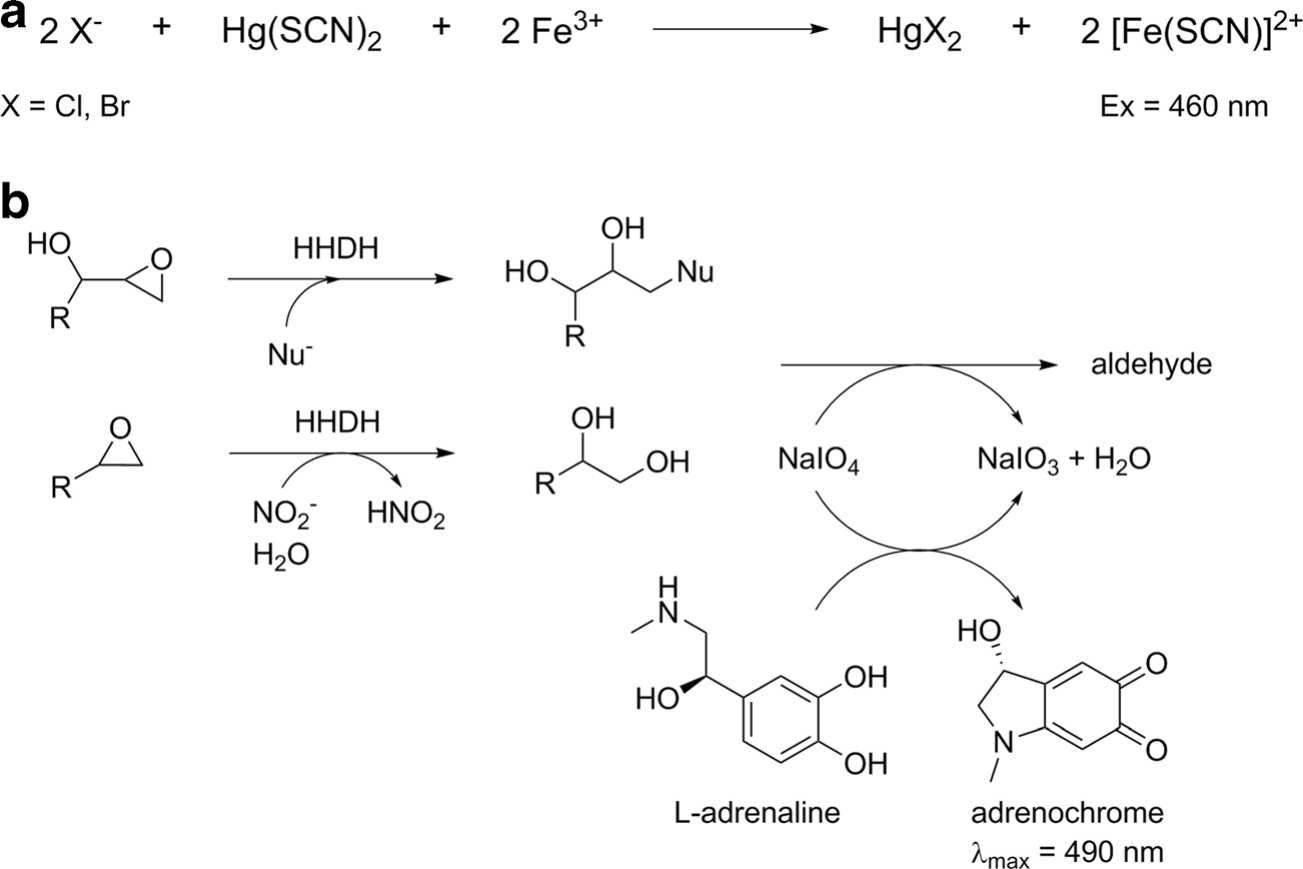
HHDH assay principles, **a** halide release assay for the detection of released chloride and bromide ions in the dehalogenation of haloalcohols (Iwasaki et al. 1952) and **b** adrenaline assay for the detection of vicinal diols formed in HHDH-catalyzed epoxide ring opening reactions (Tang et al. 2015)

Ideally, measurements of product formation rates for typical halohydrin dehalogenase reactions are commonly achieved by direct quantification of educt or product concentrations using chromatographic methods (GC, HPLC, etc.). While these methods are highly accurate, they usually require sophisticated equipment and/or can only process a limited number of samples which generally contradicts the principle of (most) protein engineering campaigns. In contrast, microtiter plate assays afford a higher throughput and several protocols have been developed for HHDH-catalyzed reactions. One of the earliest assays specifically adopted for HHDHs is the halide release assay (Bergmann and Sanik 1957). This assay is based on the detection of halide ions released by HHDHs in the dehalogenation of halohydrins (Fig. 9; Schallmey et al. 2013). Although this assay is specific, it requires the use of toxic chemicals such as mercury thiocyanate. As an alternative, a pH-based assay was reported by Tang et al. (2010), making use of phenol red as pH indicator. During HHDH-catalyzed halohydrin dehalogenation, protons are released, resulting in acidification of a weakly buffered reaction system and, thus, an absorbance change of the applied pH indicator at 560 nm. This assay was used for high-throughput screening of HHDH mutant libraries in microtiter-plate format using whole cells. Both assays, however, can only be applied in dehalogenation reactions, while HHDHs are also highly relevant for the biocatalytic production of β-substituted alcohols through epoxide ring opening. Hence, very recently, also two HHDH assays have been developed for high-throughput screening of HHDH mutant libraries in epoxide ring-opening reactions. The first one, reported by Tang et al. (2015), is based on the adrenaline test, which was previously already applied for activity screening of other enzymes such as hydrolases or Baeyer-Villiger monooxygenases (Kirschner and Bornscheuer 2008). The assay principle is based on the oxidation of periodate-sensitive compounds such as 1,2-diols or 1,2-aminoalcohols with sodium periodate. Back titration of unreacted periodate with L-adrenaline gives chromogenic adrenochrome which absorbs at 490 nm (Fig. 9). In case of HHDH catalysis, the assay can be used for epoxide ring opening of α-hydroxylated epoxides such as glycidol, which results in the formation of a vicinal diol upon ring opening with one of the accepted nucleophiles (Tang et al. 2015). Alternatively, a 1,2-diol is also generated by epoxide ring opening of any accepted epoxide substrate using nitrite or formate as nucleophile, leading to unstable esters which hydrolyze with formation of the corresponding diol (Fig. 1). Hence, the adrenaline assay for HHDHs is rather restricted in terms of epoxide substrates and nucleophiles that can be applied. The other HHDH assay reported for epoxide ring opening reactions was specifically developed for using azide as nucleophile (Wan et al. 2015a). The assay is based on the quantification of unreacted azide by adding FeCl_3_ solution, which results in formation of a blood red Fe(III)/azide complex specifically absorbing at 460 nm — an assay principle already reported in 1932 (Labruto and Randisi 1932). Now, Wan et al. (2015a) adapted and optimized this assay for the screening of a random mutagenesis library in order to identify mutants of HheA3 from *P. lavamentivorans* with improved activity in the conversion of ethyl 4-chloro-3-hydroxybutyrate into ethyl 4-azido-3-hydroxybutyrate.

Assaying the progress in cyanide-mediated epoxide ring opening reactions is highly relevant for evolving carbon-carbon bond forming activities in HHDHs. Recently, the spectrophotometric absorption at 267 nm of a specific cyanide-Ni^2+^ complex was utilized to monitor cyanide consumption in 96-well format during HHDH-catalyzed epoxide ring opening (Schallmey et al. 2015).

## Protein engineering

With proper assay protocols at hand, enzyme function can be sculpted by means of protein engineering. Until recently, only five different HHDH sequences have been known. Hence, the large majority of HHDH engineering campaigns has concentrated on only two different HHDHs, HheC and HheA2. Especially structure-guided approaches have been exceptionally successful in generating versatile HHDH variants. The thorough, ProSAR-based study of Fox et al. (2007) started from HheC wild-type and explored the accessible sequence space for variants which could render the process from ethyl (*S*)-4-chloro-3-hydroxybutyrate to ethyl (*R*)-4-cyano-3-hydroxybutyrate economically viable. Here, more than 580,000 mutants were assessed by chromatographic methods resulting in the identification of variants with at least 35 mutations that matched the required process criteria. Variant HheC-2360 —one of the enzymes originating from this study— was later examined biochemically and structurally (Schallmey et al. 2013). This HheC variant carries 37 mutations and displays a 3.1-fold increased *k*_cat_ in the dehalogenation of haloalcohol ethyl (*S*)-4-chloro-3-hydroxybutyrate, while an almost 10-fold increased *k*_cat_ in the cyanolysis of the corresponding epoxide ethyl (*S*)-3,4-epoxybutyrate is observed. At the same time, enantiopreference of the variant for the haloalcohol conversion was changed from *R* (*E*^*R*^ = 4.8 for HheC) to *S* (*E*^*S*^ = 1.9 for HheC-2360). Structural analysis of enzyme HheC-2360 revealed that few prominent changes of the enzyme active site influenced the binding of target substrate compared to wild-type HheC. Additionally, HheC-2360 exhibited an increased thermal stability of at least 8 °C due to substantial increase in buried surface area and enhanced interfacial interactions between the monomers of the tetrameric assembly.

One limitation in the biocatalytic process for the synthesis of ethyl (*R*)-4-cyano-3-hydroxybutyrate (Fig. 5) is the fact that HheC is reversibly inhibited by the ketoreductase substrate ethyl 4-chloroacetoacetate, thus hampering the development of a true one-pot cascade reaction (Ma et al. 2010). The inhibition was found to be the result of competitive binding of ethyl 4-chloroacetoacetate in the active site of HheC with a *K*_*i*_ of 0.249 μM (Chen et al. 2014). Subsequent saturation mutagenesis of residues around the active site and the substrate entrance tunnel resulted in the identification of mutant F136V + W249F, which exhibited significantly less inhibition. Nevertheless, further protein engineering would be required to generate a mutant suitable for application in a real cascade process as mutant F136V + W249F displayed also threefold lower activity toward ethyl 4-chloro-3-hydroxybutyrate compared to wild-type HheC and still significant inhibition by ethyl 4-chloroacetoacetate.

Very recently, position T134 in HheC was reported to enhance epoxide cyanolysis rates of this HHDH (Schallmey et al. 2015). By exchanging threonine 134 with alanine, a mutant was obtained that displayed up to 11-fold improved *k*_cat_ values in the cyanolysis of various tested epoxides. At the same time, this mutation also increased the dehalogenase activity of HheC significantly, while enantioselectivity was generally reduced. Interestingly, Thr134 in HheC lies in close proximity to Ser132 of the catalytic triad (Fig. 2d) and forms a hydrogen bond with the side-chain O of Ser132. Hence, mutation T134A does not directly influence nucleophile binding but rather alters the interaction between Ser132 and the substrate oxygen due to the missing hydrogen bond. Interestingly, other highly active HHDHs such as HheA3 and HheB and the majority of enzyme sequences generated by Fox et al. (2007) (e.g., HheC-2360) also lack a hydrogen bonding partner for the catalytic triad Ser.

The dehalogenase activity of HheC toward 1,3-dichloropropanol could be also improved by mutagenesis of the enzyme’s 10 C-terminal residues (Wang et al. 2015a). This C-terminal overhang was chosen for site-saturation mutagenesis as it is only present in HheC and was shown to protrude from one monomer of HheC into the active site of an opposing monomer with effects on activity and selectivity (Tang et al. 2003). Complete truncation of this C-terminal overhang (residues 246–254 in HheC) resulted in an overall lower thermostability as well as drastically reduced activity. Single site-saturation mutagenesis of these 10 residues revealed that amino acid exchanges at position W249 entailed a 2.7- to 4.3-fold higher *k*_cat_ value, while mutations at residues M252–E254 increased the enzyme’s thermostability (1.6- to 4.4-fold improved thermal inactivation half-life at 55 °C). Afterwards, combinatorial mutagenesis at positions W249 and M252-E254 gave a mutant with combined improved activity and thermostability. This resulted in the identification of a triple mutant (HheC W249P + M252L + P253D) with almost 18-fold increased thermal inactivation half-life and 4-fold improved *k*_cat_ compared to HheC wild-type. The significant increase in thermostability was concluded to be the result of two additional hydrogen bonds formed between D253 and E254. In contrast, mutation W249P disrupts a hydrogen bond between W249 and Y187, which was suggested to stabilize the conformation of the nucleophile binding pocket in HheC. In a previous study by Tang et al. (2005), it was shown that mutation Y187F also eliminated this hydrogen bond and resulted in an enhanced halide release rate for the resulting mutant. Also using 1,3-dichloropropanol as substrate, Tang and coworkers identified a number of more active HheC mutants with mutations at W139C (Tang et al. 2010) and W139V (Tang et al. 2013) later.

These authors also investigated the enantioselectivity of HheA2 (Tang et al. 2012) and HheC (Guo et al. 2015) in the dehalogenation of 2-chloro-1-phenylethanol by protein engineering. While HheC wild-type preferentially converts the (*R*)-enantiomer of this aromatic haloalcohol (with *E* = 65), wild-type HheA2 displays a low (*S*)-selectivity. Saturation mutagenesis of only three residues in HheA2 (V136, L141, and N178) resulted in mutant HheA2 N178A exhibiting largely increased (*S*)-selectivity (*E* > 200). In addition, double-mutant HheA2 V136Y + L141G was identified, which displayed an inverted enantioselectivity (*E*_*R*_ = 13) due to a lower *K*_*M*_ for (*R*)- and higher *K*_*M*_ for (*S*)-2-chloro-1-phenylethanol in comparison to the parental enzyme (Tang et al. 2012). The three residues were selected for mutagenesis after structural comparison of HheA2 and HheC, in which residues V136, L141, and N178 of HheA2 were found in equivalent positions of residues T134, W139, and N176 in HheC. The latter and four additional residues were also selected for iterative CASTing of HheC in order to generate a more (*R*)-selective as well as an (*S*)-selective variant (Guo et al. 2015). Two double mutants, HheC T134V + L142M and HheC L142F + N176H, with improved (*R*)-selectivity (*E* values of 104 and 132, respectively) toward 2-chloro-1-phenylethanol were obtained. For both, higher (*R*)-selectivity is caused by a reduced *K*_*M*_ for the (*R*)-enantiomer, while *K*_*M*_ for (*S*)-2-chloro-1-phenylethanol was significantly increased compared to wild-type HheC. The best (*S*)-selective mutant, HheC P84V + F86P + T134A + N176A, exhibited an *E* value of 101. Here too, the inverted enantioselectivity results from altered binding affinities such as higher *K*_*M*_ for (*R*)- and lower *K*_*M*_ for (*S*)-2-chloro-1-phenylethanol. At the same time, *k*_cat_ values toward both enantiomers changed significantly, resulting in dramatically altered catalytic efficiencies. While HheC wild-type displayed catalytic efficiencies of ~33,000 and 523 s^−1^ M^−1^ toward (*R*)- and (*S*)-2-chloro-1-phenylethanol, respectively, the corresponding values for the best (*S*)-selective variant were determined as 345 and ~36,000 s^−1^ M^−1^, respectively. Interestingly, mutation N178A in HheA2 (equivalent to position N176 in HheC) also conferred improved (*S*)-selectivity (see above); however, mutation N176A alone did not result in an (*S*)-selective HheC variant. Based on docking results and MD simulations, the authors could also deduce the structural basis for improved and/or inverted enantioselectivity in the identified HheC mutants.

Protein engineering of HheC was also performed in a study by Xue et al. (2015b) to increase the enzyme’s enantioselectivity in the synthesis of (*S*)-epichlorohydrin starting from prochiral 1,3-dichloropropanol. Upon single site-saturation mutagenesis of several active-site residues, mutations P175S and W249P were found to exert the desired effect. Combination of both mutations resulted in a HheC variant which produced (*S*)-epichlorohydrin in 95.3 % ee, while HheC wild-type gave only 5.2 % ee for the (*S*)-epoxide. Similarly, coworkers of Zheng obtained a more active HheC mutant for the synthesis of epichlorohydrin after introducing T134A and F186Y together with 12 other mutations in HheC (Liu et al. 2014).

Yohda and coworkers just published the first protein engineering of HheB using a combination of site-directed and random mutagenesis in order to increase its enantioselectivity in the conversion of 1,3-dichloropropanol to (*R*)-4-chloro-3-hydroxybutyronitrile via dehalogenation and subsequent cyanolysis of the intermediate epichlorohydrin (Watanabe et al. 2016). As a result, the triple mutant HheB F71W + Q125T + D199H was identified giving the final product with 98.5 % ee.

Besides the improvement of enzyme characteristics such as activity or selectivity, protein engineering can be also applied to improve the expression of heterologous proteins. In this respect, Tang et al. (2011) improved the heterologous expression of *hheA2* in *E. coli* by saturating a GTG codon at position +2. This way, variants with a CCC or CCA codon were identified displaying increased expression levels due to removal of a stable mRNA secondary structure.

## Conclusions

With the expansion of the HHDH enzyme family through sequence motif-driven database mining, four new phylogenetic HHDH subtypes D through G could be defined, with protein sequence identities between subtypes below 30 %. Although the initial biochemical and biocatalytic characterization of 17 representative novel HHDHs did not reveal any significantly new enzyme characteristics (Koopmeiners et al. 2016), the more detailed investigation of additional members of the ever growing HHDH enzyme family will likely yield enzymes with novel features in the future. This may eventually result in novel biocatalytic applications of halohydrin dehalogenases beyond the current substrate and product scopes.

So far, HheC still seems to be the only HHDH enzyme exhibiting comparably high enantioselectivity in the conversion of various substrates, whereas most other HHDHs are either unselective or display only moderate enantioselectivity toward a few selected substrates. With the help of protein engineering, however, selectivity of halohydrin dehalogenases can be easily tailored —also thanks to the availability of simple and fast screening assays— and can thus be adapted to the respective process requirements. Recent biocatalytic applications further demonstrated that (wild-type) HHDHs can be employed in reactions using high substrate loadings —a prerequisite for industrial processes— especially when applied as whole-cell biocatalysts. This will facilitate their application in future biotransformations also at larger scale.

## Electronic supplementary material

ESM 1 (PDF 445 kb)

## References

[CR1] Ager DJ, Prakash I, Schaad DR (1996). 1,2-Amino alcohols and their heterocyclic derivatives as chiral auxiliaries in asymmetric synthesis. Chem Rev.

[CR2] Barbachyn MR, Ford CW (2003). Oxazolidinone structure–activity relationships leading to linezolid. Angew Chem Int Ed.

[CR3] Bergmann JG, Sanik J (1957). Determination of trace amounts of chlorine in naphtha. Anal Chem.

[CR4] Bornscheuer UT, Hesseler M (2010). Enzymatic removal of 3-monochloropropanediol (3-MCPD) and its esters from oils. Eur J Lipid Sci Technol.

[CR5] Castro CE, Bartnicki EW (1968). Biodehalogenation. Epoxidation of halohydrins, epoxide opening, and transhalogenation by a *Flavobacterium* species. Biochemistry.

[CR6] Chen S-Y, He X-J, Wu J-P, Xu G, Yang L-R (2014). Identification of halohydrin dehalogenase mutants that resist COBE inhibition. Biotechnol Bioprocess Eng.

[CR7] Chen S-Y, Yang C-X, Wu J-P, Xu G, Yang L-R (2013). Multi-enzymatic biosynthesis of chiral β-hydroxy nitriles through co-expression of oxidoreductase and halohydrin dehalogenase. Adv Synth Catal.

[CR8] de Jong RM, Kalk KH, Tang L, Janssen DB, Dijkstra BW (2006). The X-ray structure of the haloalcohol dehalogenase HheA from *Arthrobacter* sp. strain AD2: insight into enantioselectivity and halide binding in the haloalcohol dehalogenase family. J Bacteriol.

[CR9] de Jong RM, Tiesinga JJW, Rozeboom HJ, Kalk KH, Tang L, Janssen DB, Dijkstra BW (2003). Structure and mechanism of a bacterial haloalcohol dehalogenase: a new variation of the short-chain dehydrogenase/reductase fold without an NAD(P)H binding site. EMBO J.

[CR10] de Jong RM, Tiesinga JJW, Villa A, Tang L, Janssen DB, Dijkstra BW (2005). Structural basis for the enantioselectivity of an epoxide ring opening reaction catalyzed by haloalcohol dehalogenase HheC. J Am Chem Soc.

[CR11] Dvorak P, Bidmanova S, Damborsky J, Prokop Z (2014). Immobilized synthetic pathway for biodegradation of toxic recalcitrant pollutant 1,2,3-trichloropropane. Environ Sci Technol.

[CR12] Effendi AJ, Greenaway SD, Dancer BN (2000). Isolation and characterization of 2,3-dichloro-1-propanol-degrading rhizobia. Appl Env Microbiol.

[CR13] Fox RJ, Davis SC, Mundorff EC, Newman LM, Gavrilovic V, Ma SK, Chung LM, Ching C, Tam S, Muley S, Grate J, Gruber J, Whitman JC, Sheldon RA, Huisman GW (2007). Improving catalytic function by ProSAR-driven enzyme evolution. Nat Biotechnol.

[CR14] Fuchs M, Simeo Y, Ueberbacher BT, Mautner B, Netscher T, Faber K (2009). Enantiocomplementary chemoenzymatic asymmetric synthesis of (*R*)- and (*S*)-chromanemethanol. Eur J Org Chem.

[CR15] Guo C, Chen Y, Zheng Y, Zhang W, Tao Y, Feng J, Tang L (2015). Exploring the enantioselective mechanism of halohydrin dehalogenase from *Agrobacterium radiobacter* AD1 by iterative saturation mutagenesis. Appl Environ Microbiol.

[CR16] Hasnaoui-Dijoux G, Majeric Elenkov M, Lutje Spelberg JH, Hauer B, Janssen DB (2008). Catalytic promiscuity of halohydrin dehalogenase and its application in enantioselective epoxide ring opening. ChemBioChem.

[CR17] Higgins TP, Hope SJ, Effendi AJ, Dawson S, Dancer BN (2005). Biochemical and molecular characterisation of the 2,3-dichloro-1-propanol dehalogenase and stereospecific haloalkanoic dehalogenases from a versatile *Agrobacterium* sp. Biodegradation.

[CR18] Iwasaki I, Utsumi S, Ozawa T (1952). New colorimetric determination of chloride using mercuric thiocyanate and ferric ion. Bull Chem Soc Jpn.

[CR19] Janssen DB, Dinkla IJT, Poelarends GJ, Terpstra P (2005). Bacterial degradation of xenobiotic compounds: evolution and distribution of novel enzyme activities. Environ Microbiol.

[CR20] Janssen DB, Laskin AI, Sariaslani S, Gadd GM (2007). Biocatalysis by dehalogenating enzymes. Advances in applied microbiology.

[CR21] Janssen DB, Majeric-Elenkov M, Hasnaoui G, Hauer B, Lutje Spelberg JH (2006). Enantioselective formation and ring-opening of epoxides catalysed by halohydrin dehalogenases. Biochem Soc Trans.

[CR22] Jin H-X, Hu Z-C, Liu Z-Q, Zheng Y-G (2012). Nitrite-mediated synthesis of chiral epichlorohydrin using halohydrin dehalogenase from *Agrobacterium radiobacter* AD1. Biotechnol Appl Biochem.

[CR23] Jin H-X, Liu Z-Q, Hu Z-C, Zheng Y-G (2013). Production of (*R*)-epichlorohydrin from 1,3-dichloro-2-propanol by two-step biocatalysis using haloalcohol dehalogenase and epoxide hydrolase in two-phase system. Biochem Eng J.

[CR24] Kasai N, Suzuki T, Furukawa Y (1998). Chiral C3 epoxides and halohydrins: their preparation and synthetic application. J Mol Catal B Enzym.

[CR25] Kavanagh KL, Jörnvall H, Persson B, Oppermann U (2008). Medium- and short-chain dehydrogenase/reductase gene and protein families. Cell Mol Life Sci.

[CR26] Kirschner A, Bornscheuer UT (2008). Directed evolution of a Baeyer–Villiger monooxygenase to enhance enantioselectivity. Appl Microbiol Biotechnol.

[CR27] Koopmeiners J, Halmschlag B, Schallmey M, Schallmey A (2016). Biochemical and biocatalytic characterization of 17 novel halohydrin dehalogenases. Appl Microbiol Biotechnol.

[CR28] Labruto G, Randisi D (1932) Ann Chim Appl 319–324

[CR29] Liu Z-Q, Gao A-C, Wang Y-J, Zheng Y-G, Shen Y-C (2014). Expression, characterization, and improvement of a newly cloned halohydrin dehalogenase from *Agrobacterium tumefaciens* and its application in production of epichlorohydrin. J Ind Microbiol Biotechnol.

[CR30] Löytynoja A, Goldman N (2010). webPRANK: a phylogeny-aware multiple sequence aligner with interactive alignment browser. BMC Bioinformatics.

[CR31] Lutje Spelberg JH, Tang L, Kellogg RM, Janssen DB (2004). Enzymatic dynamic kinetic resolution of epihalohydrins. Tetrahedron Asymmetry.

[CR32] Lutje Spelberg JH, van Hylckama Vlieg JET, Bosma T, Kellogg RM, Janssen DB (1999). A tandem enzyme reaction to produce optically active halohydrins, epoxides and diols. Tetrahedron Asymmetry.

[CR33] Ma SK, Gruber J, Davis C, Newman L, Gray D, Wang A, Grate J, Huisman GW, Sheldon RA (2010). A green-by-design biocatalytic process for atorvastatin intermediate. Green Chem.

[CR34] Majeric Elenkov M, Hoeffken HW, Tang L, Hauer B, Janssen DB (2007). Enzyme-catalyzed nucleophilic ring opening of epoxides for the preparation of enantiopure tertiary alcohols. Adv Synth Catal.

[CR35] Majeric Elenkov M, Primožič I, Hrenar T, Smolko A, Dokli I, Salopek-Sondi B, Tang L (2012). Catalytic activity of halohydrin dehalogenases towards spiroepoxides. Org Biomol Chem.

[CR36] Majeric Elenkov M, Tang L, Meetsma A, Hauer B, Janssen DB (2008). Formation of enantiopure 5-substituted oxazolidinones through enzyme-catalysed kinetic resolution of epoxides. Org Lett.

[CR37] Mikleusevic A, Hamersak Z, Salopek-Sondi B, Tang L, Janssen DB, Majeric Elenkov M (2015). Oxazolidinone synthesis through halohydrin dehalogenase-catalyzed dynamic kinetic resolution. Adv Synth Catal.

[CR38] Nakamura T, Yu F, Mizunashi W, Watanabe I (1991). Microbial transformation of prochiral 1,3-dichloro-2-propanol into optically-active 3-chloro-1,2-propanediol. Agric Biol Chem.

[CR39] Poelarends GJ, van Hylckama Vlieg JET, Marchesi JR, Freitas Dos Santos LM, Janssen DB (1999). Degradation of 1,2-dibromoethane by *Mycobacterium* sp. strain GP1. J Bacteriol.

[CR40] Schallmey M, Floor RJ, Hauer B, Breuer M, Jekel PA, Wijma HJ, Dijkstra BW, Janssen DB (2013). Biocatalytic and structural properties of a highly engineered halohydrin dehalogenase. ChemBioChem.

[CR41] Schallmey M, Floor RJ, Szymanski W, Janssen DB, Carreira EM, Yamamoto H (2012). 7.8 Hydrolysis and reverse hydrolysis: halohydrin dehalogenases. Comprehensive chirality.

[CR42] Schallmey M, Jekel P, Tang L, Majerić Elenkov M, Höffken HW, Hauer B, Janssen DB (2015). A single point mutation enhances hydroxynitrile synthesis by halohydrin dehalogenase. Enzyme Microb Technol.

[CR43] Schallmey M, Koopmeiners J, Wells E, Wardenga R, Schallmey A (2014). Expanding the halohydrin dehalogenase enzyme family: identification of novel enzymes by database mining. Appl Environ Microbiol.

[CR44] Schrittwieser JH, Lavandera I, Seisser B, Mautner B, Kroutil W (2009). Biocatalytic cascade for the synthesis of enantiopure β-azidoalcohols and β-hydroxynitriles. Eur J Org Chem.

[CR45] Szymanski W, Postema CP, Tarabiono C, Berthiol F, Campbell-Verduyn L, de Wildeman S, de Vries JG, Feringa BL, Janssen DB (2010). Combining designer cells and click chemistry for a one-pot four-step preparation of enantiopure β-hydroxytriazoles. Adv Synth Catal.

[CR46] Tang L, Jiang R, Zheng K, Zhu X (2011). Enhancing the recombinant protein expression of halohydrin dehalogenase HheA in *Escherichia coli* by applying a codon optimization strategy. Enzyme Microb Technol.

[CR47] Tang L, Li Y, Wang X (2010). A high-throughput colorimetric assay for screening halohydrin dehalogenase saturation mutagenesis libraries. J Biotechnol.

[CR48] Tang L, Liu Y, Jiang R, Zheng Y, Zheng K, Zheng H (2015). A high-throughput adrenaline test for the exploration of the catalytic potential of halohydrin dehalogenases in epoxide ring-opening reactions. Biotechnol Appl Biochem.

[CR49] Tang L, Torres Pazmino DE, Fraaije MW, de Jong RM, Dijkstra BW, Janssen DB (2005). Improved catalytic properties of halohydrin dehalogenase by modification of the halide-binding site. Biochemistry.

[CR50] Tang L, van Merode AEJ, Lutje Spelberg JH, Fraaije MW, Janssen DB (2003). Steady-state kinetics and tryptophan fluorescence properties of halohydrin dehalogenase from *Agrobacterium radiobacter*. Roles of W139 and W249 in the active site and halide-induced conformational change. Biochemistry.

[CR51] Tang L, Zheng K, Liu Y, Zheng H, Wang H, Song C, Zhou H (2013). Exploring the potential of megaprimer PCR in conjunction with orthogonal array design for mutagenesis library construction. Biotechnol Appl Biochem.

[CR52] Tang L, Zhu X, Zheng H, Jiang R, Elenkov MM (2012). Key residues for controlling enantioselectivity of halohydrin dehalogenase from *Arthrobacter* sp. strain AD2, revealed by structure-guided directed evolution. Appl Environ Microbiol.

[CR53] Trifinopoulos J, Nguyen L-T, von Haeseler A, Minh BQ (2016) W-IQ-TREE: a fast online phylogenetic tool for maximum likelihood analysis. Nucleic Acids Res. gkw256. doi: 10.1093/nar/gkw256 10.1093/nar/gkw256 PMC4987875 27084950

[CR54] van den Wijngaard AJ, Janssen DB, Witholt B (1989). Degradation of epichlorohydrin and halohydrins by bacterial cultures isolated from freshwater sediment. J Gen Microbiol.

[CR55] van Hylckama Vlieg JET, Tang L, Lutje Spelberg JH, Smilda T, Poelarends GJ, Bosma T, van Merode AEJ, Fraaije MW, Janssen DB (2001). Halohydrin dehalogenases are structurally and mechanistically related to short-chain dehydrogenases/reductases. J Bacteriol.

[CR56] Wan N-W, Liu Z-Q, Huang K, Shen Z-Y, Xue F, Zheng Y-G, Shen Y-C (2014). Synthesis of ethyl (*R*)-4-cyano-3-hydroxybutyrate in high concentration using a novel halohydrin dehalogenase HHDH-PL from *Parvibaculum lavamentivorans* DS-1. RSC Adv.

[CR57] Wan N-W, Liu Z-Q, Xue F, Huang K, Tang L-J, Zheng Y-G (2015). An efficient high-throughput screening assay for rapid directed evolution of halohydrin dehalogenase for preparation of β-substituted alcohols. Appl Microbiol Biotechnol.

[CR58] Wan N-W, Liu Z-Q, Xue F, Shen Z-Y, Zheng Y-G (2015). A one-step biocatalytic process for (*S*)-4-chloro-3-hydroxybutyronitrile using halohydrin dehalogenase: a chiral building block for atorvastatin. ChemCatChem.

[CR59] Wan N-W, Liu Z-Q, Xue F, Zheng Y-G (2015). An enzymatic method for determination of azide and cyanide in aqueous phase. J Biotechnol.

[CR60] Wang X, Han S, Yang Z, Tang L (2015). Improvement of the thermostability and activity of halohydrin dehalogenase from *Agrobacterium radiobacter* AD1 by engineering C-terminal amino acids. J Biotechnol.

[CR61] Wang X, Lin H, Zheng Y, Feng J, Yang Z, Tang L (2015). MDC-analyzer-facilitated combinatorial strategy for improving the activity and stability of halohydrin dehalogenase from *Agrobacterium radiobacter* AD1. J Biotechnol.

[CR62] Watanabe F, Yu F, Ohtaki A, Yamanaka Y, Noguchi K, Yohda M, Odaka M (2015). Crystal structures of halohydrin hydrogen-halide-lyases from *Corynebacterium* sp. N-1074. Proteins Struct Funct Bioinforma.

[CR63] Watanabe F, Yu F, Ohtaki A, Yamanaka Y, Noguchi K, Odaka M, Yohda M (2016). Improvement of enantioselectivity of the B-type halohydrin hydrogen-halide lyase from *Corynebacterium* sp. N-1074. J Biosci Bioeng.

[CR64] Xue F, Liu Z-Q, Wan N-W, Zheng Y-G (2014). Purification, gene cloning, and characterization of a novel halohydrin dehalogenase from *Agromyces mediolanus* ZJB120203. Appl Biochem Biotechnol.

[CR65] Xue F, Liu Z-Q, Wang Y-J, Wan N-W, Zheng Y-G (2015). Biochemical characterization and biosynthetic application of a halohydrin dehalogenase from *Tistrella mobilis* ZJB1405. J Mol Catal B Enzym.

[CR66] Xue F, Liu Z-Q, Wang Y-J, Zhu H-Q, Wan N-W, Zheng Y-G (2015). Efficient synthesis of (*S*)-epichlorohydrin in high yield by cascade biocatalysis with halohydrin dehalogenase and epoxide hydrolase mutants. Catal Commun.

[CR67] Yao P, Wang L, Yuan J, Cheng L, Jia R, Xie M, Feng J, Wang M, Wu Q, Zhu D (2015). Efficient biosynthesis of ethyl (*R*)-3-hydroxyglutarate through a one-pot bienzymatic cascade of halohydrin dehalogenase and nitrilase. ChemCatChem.

[CR68] You Z-Y, Liu Z-Q, Zheng Y-G (2012). Properties and biotechnological applications of halohydrin dehalogenases: current state and future perspectives. Appl Microbiol Biotechnol.

[CR69] Yu F, Nakamura T, Mizunashi W, Watanabe I (1994). Cloning of two halohydrin hydrogen-halide-lyase genes of *Corynebacterium* sp. strain N-1074 and structural comparison of the genes and gene products. Biosci Biotechnol Biochem.

[CR70] Zou S-P, Du E-H, Hu Z-C, Zheng Y-G (2013). Enhanced biotransformation of 1,3-dichloro-2-propanol to epichlorohydrin via resin-based in situ product removal process. Biotechnol Lett.

[CR71] Zou S-P, Zheng Y-G, Du E-H, Hu Z-C (2014). Enhancement of (*S*)-2,3-dichloro-1-propanol production by recombinant whole-cell biocatalyst in *n*-heptane–aqueous biphasic system. J Biotechnol.

