# Supplementary material for: Recent advances on halohydrin dehalogenases—from enzyme identification to novel biocatalytic applications
Source: Appl Microbiol Biotechnol. 2016 Aug 8;100:7827–39. doi: 10.1007/s00253-016-7750-y (PMC4989007; doi:10.1007/s00253-016-7750-y)
Supplement: Supplementary file 1 — (PDF 445 kb) [file 253_2016_7750_MOESM1_ESM.pdf]

Applied Microbiology and Biotechnology

Supplemental Material to

Recent advances on halohydrin dehalogenases – from enzyme identification to novel biocatalytic applications

Anett Schallmey* and Marcus Schallmey

Affiliation:
Technische Universität Braunschweig
Institut für Biochemie, Biotechnologie und Bioinformatik
Spielmannstr. 7
38106 Braunschweig
Germany

* Corresponding author:
Email: a.schallmey@tu-braunschweig.de
Phone: +49 531 391-55400
Fax: +49 531 391-55401

Table S1. Halohydrin dehalogenases (HHDHs) of the A- through G-subtype identified by motif-based database mining in GenBank (release 213).

**HHDHs**	**Source**	**Accession**	**Experimentally verified in**
HheA	*Corynebacterium* sp. N-1074	BAA14361	Yu et al. 1994
HheA2	*Arthrobacter* sp. AD2	AAK92100	van Hylckama Vlieg et al. 2001
HheA3	*Parvibaculum lavamentivorans* DS-1	WP_012111877	Wan et al. 2014; Schallmey et al. 2014; Koopmeiners et al. 2016
HheA4	*Arthrobacter* sp. JBH1	WP_028708016	
HheA5	*Tistrella mobilis* KA081020-065	WP_014743557	Schallmey et al. 2014; Xue et al. 2015; Koopmeiners et al. 2016
HheA6	*Candidatus Phaeomarinobacter ectocarpi* Ec32	WP_052534782	
HheA7	*Sneathiella glossodoripedis* JCM 23214	WP_025899379	
HheA8	alpha proteobacterium Mf 1.05b.01	WP_051402546	
HheA9	*Tepidicaulis marinus* MA2	WP_052379152	
HheA10	*Tsukamurella* sp. 1534	WP_019201195	
HheA11	*Reyranella massiliensis* 521	WP_020698933	unpublished data
HheA12	*Agromyces mediolanus* ZJB 120203	AGL34059	Xue et al. 2014
HheA13	*Pseudomonas* sp. G5(2012)	WP_042955870	
HheA14	*Streptomyces showdoensis*	WP_046910876	
HheA15	*Tistrella mobilis* MCCC 1A02139	WP_062762711	
HheB	*Corynebacterium* sp. N-1074	BAA14362	Yu et al. 1994
HheB2	*Mycobacterium* sp. GP1	AAK73175	van Hylckama Vlieg et al. 2001
HheB3	marine metagenome	EBL02020	Koopmeiners et al. 2016
HheB4	marine metagenome	EBP61646	Schallmey et al. 2014; Koopmeiners et al. 2016
HheB5	marine metagenome	ECR06649	Schallmey et al. 2014; Koopmeiners et al. 2016
HheB6	marine metagenome	EDB56284	Schallmey et al. 2014; Koopmeiners et al. 2016
HheB7	marine metagenome	EDD65701	Schallmey et al. 2014; Koopmeiners et al. 2016
HheC	*Agrobacterium tumefaciens* AD1	AAK92099	van Hylckama Vlieg et al. 2001
HheD	*Dechloromonas aromatica* RCB	WP_011285856	Schallmey et al. 2014; Koopmeiners et al. 2016
HheD2	gamma proteobacterium HTCC2207	WP_007233072	Schallmey et al. 2014; Koopmeiners et al. 2016
HheD3	*Methylibium petroleiphilum* PM1	WP_011828277	Schallmey et al. 2014; Koopmeiners et al. 2016
HheD4	marine metagenome	ECY18578	Schallmey et al. 2014
HheD5	*Thauera* sp. MZ1T	WP_012585440	Schallmey et al. 2014; Koopmeiners et al. 2016
HheD6	*Marinobacter nanhaiticus* D15-8W	WP_004579485	Schallmey et al. 2014
HheD7	*Thauera* sp. 27	WP_002926105	
HheD8	*Thauera aminoaromatica* S2	WP_004302136	
HheD9	*Thauera phenylacetica* B4P	WP_004355811	
HheD10	*Limnohabitans* sp. Rim28	WP_040506033	
HheD11	*Thiothrix disciformis* DSM 14473	WP_020394200	
HheD12	*Pseudomonas pelagia* CL-AP6	WP_022962804	
HheD13	*Betaproteobacteria* bacterium MOLA814	WP_023472742	
HheD14	*Gammaproteobacteria* bacterium MOLA455	WP_035490777	
HheD15	*Candidatus Competibacter denitrificans* Run_A_D11	WP_048670059	
HheD16	*Methylibium* sp. T29	WP_036236554	
HheD17	*Curvibacter gracilis* ATCC BAA-807	WP_051443321	
HheD18	*Curvibacter lanceolatus* ATCC 14669	WP_051107362	
HheD19	*Idiomarina salinarum* ISL-52	WP_034774738	

HheD20	*Rhodocyclaceae* bacterium PG1-Ca6	AJP48347	
HheD21	*Desulfatitalea* sp. BRH_c12	KJS31884	
HheD22	marine sediment metagenome	KKN91043	
HheD23	marine sediment metagenome	KKN99199	
HheD24	marine sediment metagenome	KKO03046	
HheD25	*Ideonella sakaiensis* 201-F6	GAP36263	
HheD26	*Limnohabitans planktonicus* II-D5	WP_053169862	
HheD27	*Desulfatitalea tepidiphila* S28bF	WP_054029282	
HheD28	beta proteobacterium AAP51	WP_054155488	
HheD29	beta proteobacterium AAP65	WP_054139362	
HheD30	*Limnohabitans* sp. 63ED37-2	ALK88134	
HheD31	*Methylibium* sp. Root1272	WP_056325236	
HheD32	SAR92 bacterium BACL16 MAG-120619-bin48	KRP19042	
HheD33	SAR92 bacterium BACL16 MAG-120322-bin99	KRP26526	
HheD34	*Noviherbaspirillum* sp. Root189	WP_057289066	
HheD35	*Pseudohongiella spirulinae*	WP_058022908	
HheD36	*Pseudacidovorax intermedius* NS331	WP_058643388	
HheD37	*Halioglobus* sp. HI00S01	KZX60518	
HheD38	*Hydrogenophaga* sp. LPB0072	OAD40436	
HheE	marine metagenome	EBP63112	Schallmey et al. 2014; Koopmeiners et al. 2016
HheE2	marine metagenome	ECW41905	Schallmey et al. 2014; Koopmeiners et al. 2016
HheE3	marine metagenome	EDF62577	Schallmey et al. 2014; Koopmeiners et al. 2016
HheE4	marine metagenome	EDH34310	Schallmey et al. 2014
HheE5	gamma proteobacterium IMCC3088	WP_009577001	Schallmey et al. 2014; Koopmeiners et al. 2016
HheF	uncultured bacterium	BAH89601	Schallmey et al. 2014; Koopmeiners et al. 2016
HheG	*Ilumatobacter coccineus* YM16-304	WP_015443096	Schallmey et al. 2014; Koopmeiners et al. 2016
HheG2	*Ilumatobacter nonamiensis* YM16-303	WP_040495182	unpublished data

References

Koopmeiners J, Halmschlag B, Schallmey M, Schallmey A (2016) Biochemical and biocatalytic characterization of 17 novel halohydrin dehalogenases. Appl Microbiol Biotechnol 1–11. doi: 10.1007/s00253-016-7493-9

Schallmey M, Koopmeiners J, Wells E, Wardenga R, Schallmey A (2014) Expanding the halohydrin dehalogenase enzyme family: Identification of novel enzymes by database mining. Appl Environ Microbiol 80:7303–7315. doi: 10.1128/AEM.01985-14

van Hylckama Vlieg JET, Tang L, Lutje Spelberg JH, Smilda T, Poelarends GJ, Bosma T, van Merode AEJ, Fraaije MW, Janssen DB (2001) Halohydrin dehalogenases are structurally and mechanistically related to short-chain dehydrogenases/reductases. J Bacteriol 183:5058–5066. doi: 10.1128/JB.183.17.5058-5066.2001

Wan N-W, Liu Z-Q, Huang K, Shen Z-Y, Xue F, Zheng Y-G, Shen Y-C (2014) Synthesis of ethyl (*R*)-4-cyano-3-hydroxybutyrate in high concentration using a novel halohydrin dehalogenase HHDH-PL from *Parvibaculum lavamentivorans* DS-1. RSC Adv 4:64027–64031. doi: 10.1039/C4RA13646B

Xue F, Liu Z-Q, Wan N-W, Zheng Y-G (2014) Purification, gene cloning, and characterization of a novel halohydrin dehalogenase from *Agromyces mediolanus* ZJB120203. Appl Biochem Biotechnol 174:352–364. doi: 10.1007/s12010-014-1111-z

Xue F, Liu Z-Q, Wang Y-J, Wan N-W, Zheng Y-G (2015) Biochemical characterization and biosynthetic application of a halohydrin dehalogenase from *Tistrella mobilis* ZJB1405. J Mol Catal B Enzym 115:105–112. doi: 10.1016/j.molcatb.2015.02.008

Yu F, Nakamura T, Mizunashi W, Watanabe I (1994) Cloning of two halohydrin hydrogen-halide-lyase genes of *Corynebacterium* sp. strain N-1074 and structural comparison of the genes and gene products. Biosci Biotechnol Biochem 58:1451–1457.
